# Blade needle therapy versus conventional acupuncture for knee osteoarthritis: A meta-analysis

**DOI:** 10.1097/MD.0000000000029647

**Published:** 2022-07-29

**Authors:** Xunlong Yin, Yuan Liu, Wu Liu, Wei Liang, Qingyong Liang

**Affiliations:** Guangxi University of Chinese Medicine, Nanning, China.

**Keywords:** blade needle, conventional acupuncture, knee osteoarthritis, meta-analysis

## Abstract

**Background:**

This study investigated the hypothesis that the efficacy of blade needle therapy for the treatment of knee osteoarthritis (KOA) is superior to that of conventional acupuncture. In addition, the efficacy of blade needle therapy versus conventional acupuncture for the treatment of KOA was analyzed in a meta-analysis.

**Methods:**

Randomized controlled trials (RCTs) of blade needle therapy and conventional acupuncture for treating KOA were retrieved from the electronic databases CNKL, Wanfang, VIP, PubMed, EMBASE and the Cochrane Library from the commencement of each database to July of 2021. Data were extracted and evaluated by 2 reviewers independently. RevMan 5.3 software was used to conduct the meta-analysis after the studies were evaluated.

**Results:**

A total of 11 RCTs were included, all from China, involving 1142 patients. The meta-analysis results showed that the effective rate of the blade needle group was better than that of the conventional acupuncture group (OR = 3.61, 95% CI [2.56–5.10], *P* < .00001).

**Conclusion:**

The efficacy of blade needle treatment for KOA is superior to that of conventional acupuncture, but more high-quality studies are needed for future validation due to the low proportion of high-quality studies included and the possible bias factor.

## 1. Introduction

Knee osteoarthritis (KOA) is a common and complex disease affecting approximately 10% of the global population over 60 years of age.^[1]^ In Europe, approximately 25% of people aged over 55 have a previous record of knee pain, and a quarter of patients may become disabled as a result.^[2]^ Approximately 37% of people over the age of 60 in the United States have KOA.^[3]^ In China, the prevalence of KOA is nearly 50% in people above age 60, 53% of patients are disabled and live with difficulty, and more than 65% of patients have recurrent attacks that persist, seriously affecting their survival quality.^[4–6]^ However, the treatment outcome is hardly satisfactory for patients or medical practitioners and has become an urgent problem for clinicians.^[7]^ Neither nonsteroidal anti-inflammatory drugs (NSAIDs) nor intra-articular injections of sodium glacial acetic acid for KOA seem to cause adverse effects such as gastric bleeding, peptic ulcers and allergies.^[8,9]^ Although surgery is the last resort, it is often ineffective and associated with numerous surgical complications^[10–15]^; therefore, the search for a more effective treatment is one of the current hot topics of research in the treatment of KOA. Conventional acupuncture therapy is one of the characteristic therapies of traditional Chinese medicine (TCM) osteopathy, which not only facilitates the reduction of inflammation but also promotes blood circulation in local tissues.^[16]^ In recent years, conventional acupuncture therapy has been extensively used clinically for its safety and effectiveness, and reports on acupotomy and acupuncture for KOA treatment are increasing.^[17–21]^ Acupotomy not only loosens and peels away adherent tissues but also stimulates acupoints to produce a meridian sensing effect.^[22]^ Acupuncture treatment for KOA reduces pain and morning stiffness in the diseased area, slows degenerative changes in articular cartilage and restores knee function,^[23,24]^ which improves the efficacy and shortens the course of treatment.^[25]^ Recently, a new type of external TCM blade needle that incorporates the characteristics of acupotomy and acupuncture has been applied.^[26–28]^ Studies on the efficacy of blade needle treatment for KOA have been increasing, but a review of the literature has not provided a systematic and objective evaluation of the efficacy of blade needle treatment for KOA. Therefore, in this study, an objective and credible meta-analysis was conducted to provide a reliable basis for the clinical treatment of KOA by reviewing a large number of relevant studies and to report the status of the use of blade needle in clinical practice and the problems that exist. The analysis is as follows.

## 2. Materials and Methods

Statement: This study is a meta-analysis, which is primarily a process of collecting data and analyzing them with statistical software to draw conclusions. This study is not about human or animal experiments and this study does not involve ethical issues.

### 2.1. Search strategy

The search databases included the China National Knowledge Infrastructure, Wanfang Data, VIP, PubMed, EMBASE, Cochrane Library, etc. All the databases were searched from their origin to July 25, 2021, and only studies in Chinese or English were searched.

The Chinese database search formula was as follows: (“Knee Osteoarthritis” OR “Knee Osteoarthritides” OR “Osteoarthritis of Knee” OR “Osteoarthritis of the Knee” AND (“Blade needle” OR “Edge needle” OR “Cutting needle” OR “needle-knife” OR “acupotomology” OR “acupotome” OR “acupotomy”) AND (“Random”). The English database search formula was as follows: (“Knee Osteoarthritis” OR “Knee Osteoarthritides” OR “Osteoarthritis of Knee” OR “Osteoarthritis of the Knee”) AND (“Blade needle” OR “Edge needle” OR “Cutting needle” OR “needle-knife” OR “acupotomology” OR “acupotome” OR “acupotomy”) AND (“Random”).

### 2.2. Literature inclusion criteria

(1) Study type: Randomized controlled trials (RCTs) or semirandomized controlled trials of blade needle versus conventional acupuncture in the treatment of KOA, limited to Chinese and English, were collected. (2) Study population: Studies of patients with KOA, regardless of the source, wherein patient age and sex and clear and recognized diagnostic criteria were reported were included. (3) Interventions: Blade needle therapy was performed in the treatment group (not combined with other therapies), and conventional acupuncture was performed in the control group (excluding electroacupuncture); studies of combinations of other therapies were excluded. (4) Outcome index: Studies reporting a treatment efficiency outcome were included.

### 2.3. Literature exclusion criteria

The exclusion criteria were as follows: (1) nonRCTs, (2) studies with unclear or nonaccepted diagnostic criteria or efficacy criteria, (3) studies that included other therapies, such as manipulation, moxibustion, and drugs, (4) animal experimental studies, and (5) theoretical and review literature and repeatedly published literature.

### 2.4. Data extraction and literature quality evaluation

Two evaluators independently screened the literature and first read the title and abstract of the literature to initially exclude those studies that did not meet the inclusion criteria; if they could not judge, they read the full text to further judge; if 2 independent evaluators disagreed on the inclusion or noninclusion of the literature and the quality score, the 2 evaluators sought a mutually agreeable third evaluator to discuss and negotiate a conclusion.

The included literature was evaluated using the risk of bias assessment tool recommended by the Cochrane Handbook for Systematic Reviews, which includes seven entries: generation of randomized sequences; allocation concealment of randomized protocols; blinding of study subjects and performers; blinding of outcome evaluators; completeness of outcome data; selective reporting; and other sources of bias. Each entry was classified into 3 levels: low risk, unclear risk, and high risk of bias.

The main information extracted included (1) information related to the included studies, such as the title of the literature, first author, and time of publication; (2) information related to the study population, including number of patients, age, follow-up time, and intervention modality; and (3) each outcome indicator of interest.

### 2.5. Statistical analysis of data

RevMan 5.3 software provided by the Cochrane Collaboration Network was used for the analysis. Due to the different data units reported in the original literature, continuous variables were used using standardized mean difference (SMD) representation, and estimates and 95% confidence intervals (CIs) are given for each effect indicator.

The *χ*^2^ test and Higgins I^2^ test were used to test for heterogeneity among the outcomes, and if I2 ≤ 50%, a fixed-effects model was used for analysis; if 50%<I2 ≤ 75%, a random-effects model was used for analysis; I2 > 75% was not included in the combined analysis.

If > 10 studies were included, Egger test and funnel plots were used to detect publication bias. *P* > 0.05 indicates nonsignificant publication bias, and *P* < 0.05 indicates publication bias. The test level for the meta-analysis was set at a = 0.05.

## 3. Results

**Figure 1.**
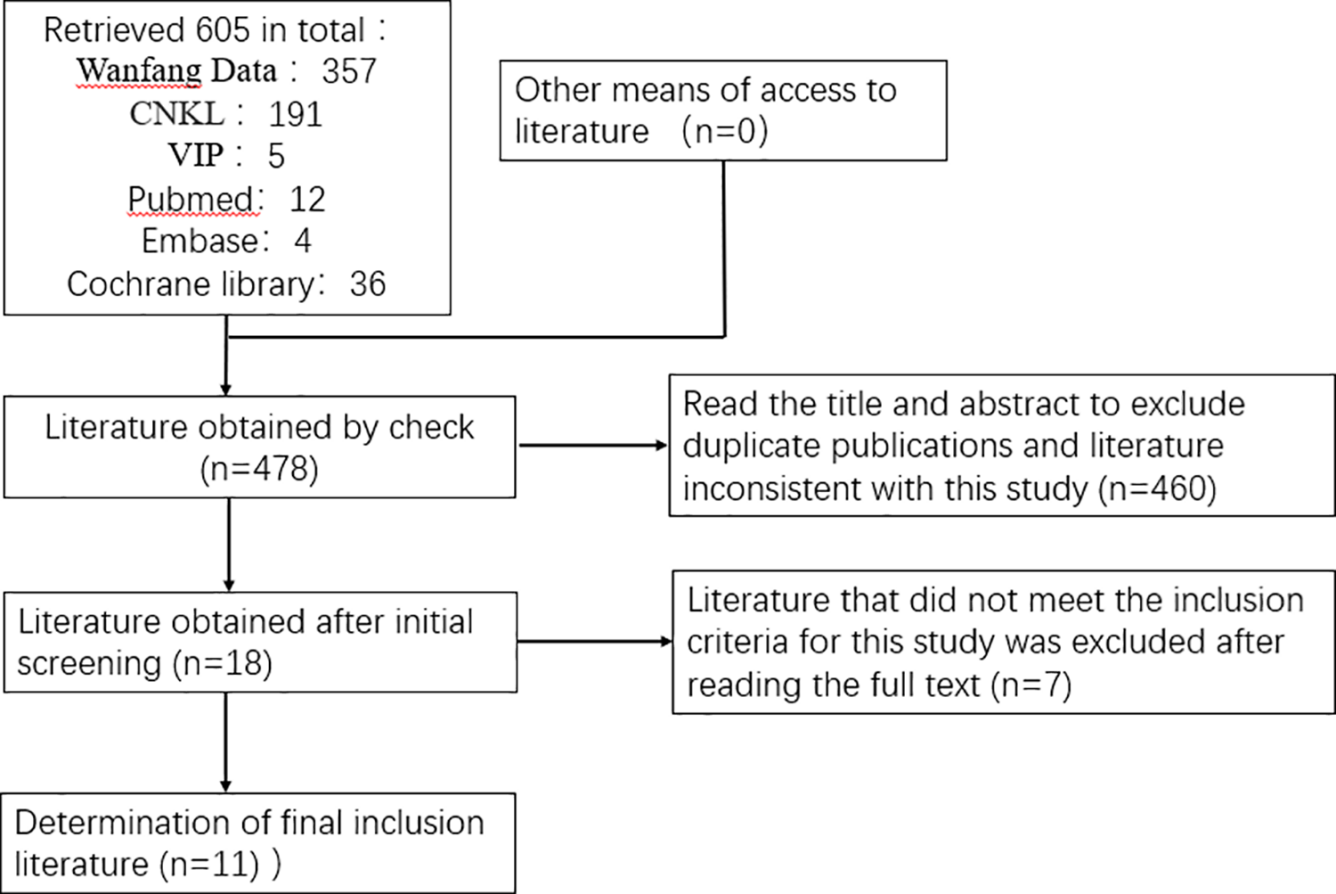
Flow chart of literature screening.

**Figure 2.**
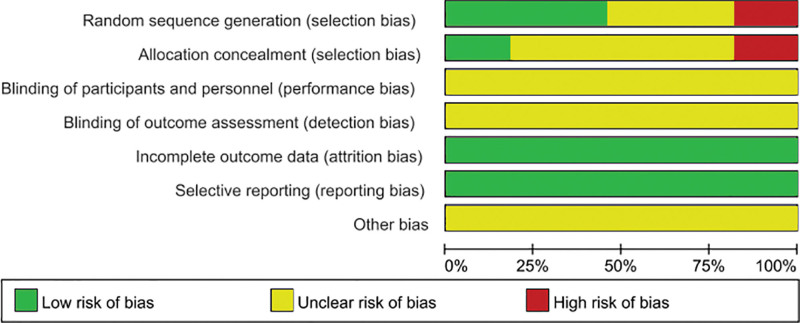
Risk of bias summary.

**Figure 3.**
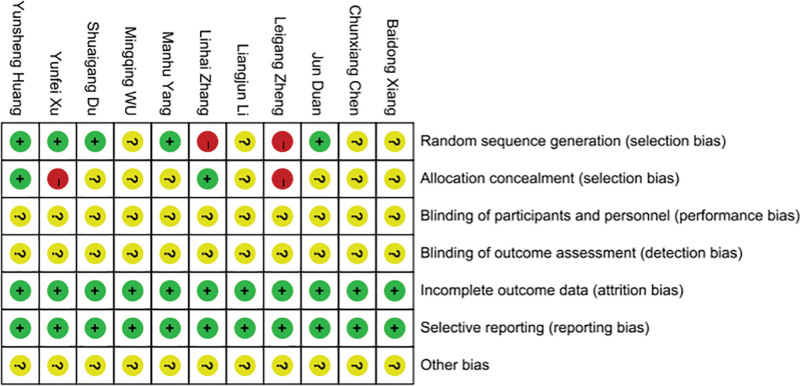
Risk of bias summary.

A total of 605 documents were retrieved from various databases based on the search strategy. After checking and reading the titles and abstracts, 478 studies were initially screened. After reading the full text, studies that did not meet the inclusion criteria were excluded, and 11 articles remained for inclusion in the meta-analysis,^[29–39]^ all of which were published in Chinese. The literature screening process is shown in Figure 1. Eleven publications involving a total of 1142 KOA patients were included, with 571 in the trial group and 571 in the control group. In the test group, 8 were treated with a blade needle, 2 with a curved blade needle, and 1 with an oblique circular blade needle. In the control group, all were treated with conventional acupuncture. The basic characteristics of the specific included studies are shown in Table 1. The risk of bias for the included trials is summarized in Figures 2 and 3.

**Table 1:** Characteristics of included articles.

Name	Published time	The number of cases		Measure		Period of treatment	Lost/quit
		Blade needle group	Conventional acupuncture group	Blade needle group	Conventional acupuncture group		
Baidong Xiang	2019	36	36	Blade needle	Conventional acupuncture	10 days	No
Chunxiang Chen	2018	58	58	Blade needle	Conventional acupuncture	3 weeks	No
Jun Duan	2014	30	30	Blade needle	Conventional acupuncture	3 weeks	No
Leigang Zheng	2014	128	130	Blade needle	Conventional acupuncture	2 weeks	Yes
Liangjun Li	2013	50	50	Blade needle	Conventional acupuncture	3 weeks	No
Linhai Zhang	2018	34	34	Blade needle	Conventional acupuncture	4 weeks	No
Manhu Yang	2020	31	31	Blade needle	Conventional acupuncture	3 weeks	No
Mingqing Wu	2011	50	50	Blade needle	Conventional acupuncture	3 weeks	No
Shuaigang Du	2018	74	72	Blade needle	Conventional acupuncture	4 weeks	Yes
Yunfei Xu	2019	50	50	Blade needle	Conventional acupuncture	2 weeks	No
Yunsheng Huang	2015	30	30	Blade needle	Conventional acupuncture	2 weeks	Yes

**Figure 4.**
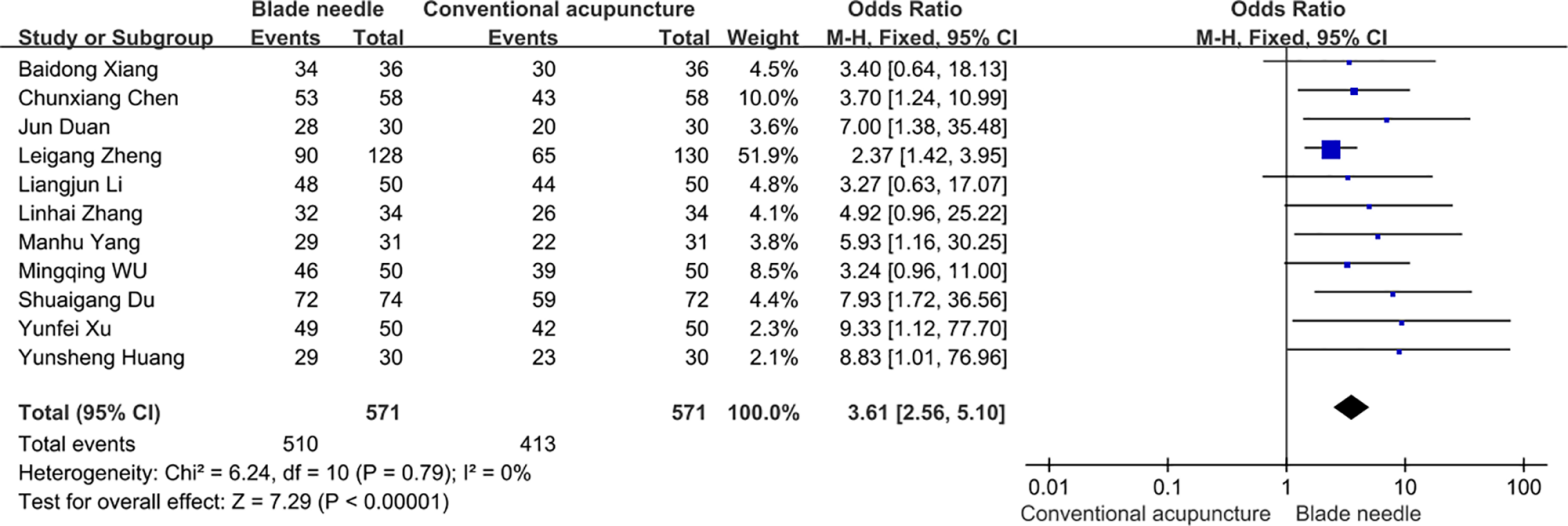
Clinical efficiency of blade acupuncture versus conventional acupuncture in the treatment of osteoarthritis of the knee.

**Figure 5.**
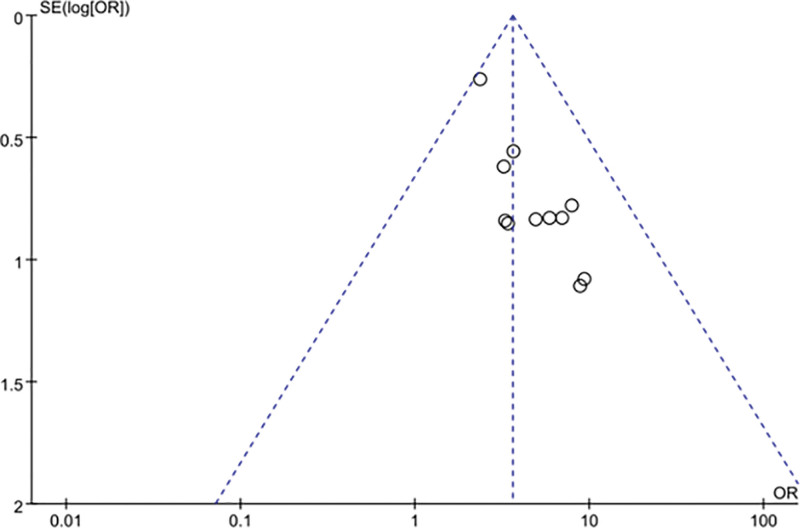
Clinical effectiveness funnel chart.

The clinical efficacy of the blade needle group was analyzed in comparison with that of the conventional acupuncture group, and the results of the heterogeneity test were *χ*^2^ = 6.24, *P* = .79, I2 = 0%. This finding indicates that there was no significant heterogeneity between the studies using a fixed effects model. The results of the meta-analysis showed statistically significant differences in efficacy between the blade needle group and the conventional acupuncture group (OR = 3.61, 95% CI [2.56, 5.10], Z = 7.29, *P* < .00001). Blade acupuncture had significantly higher efficacy than conventional acupuncture for the treatment of KOA (Fig. 4). The clinical efficacy funnel plot showed poor symmetry, suggesting possible publication bias in the literature included in the analysis (Fig. 5).

**Figure 6.**
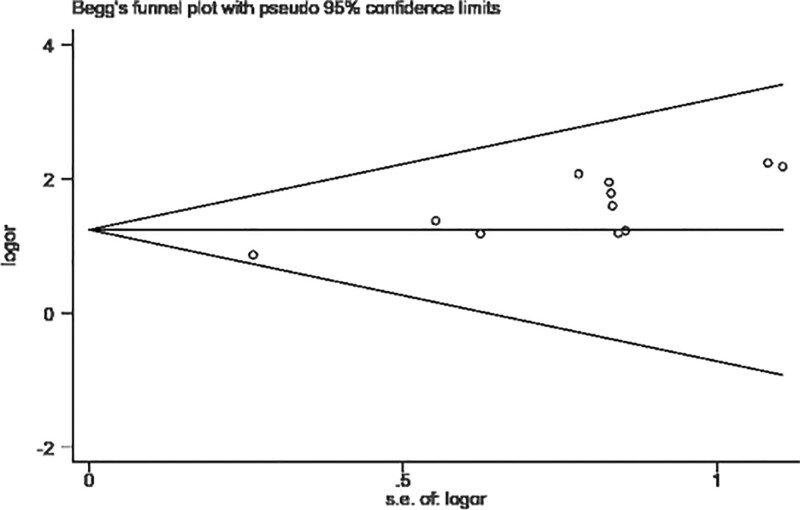
Begg’s test.

**Figure 7.**
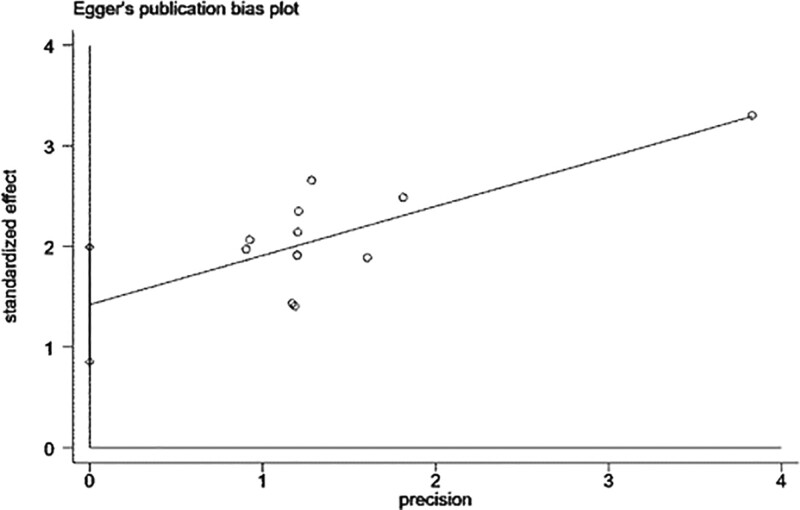
Egger test.

To further test whether there was publication bias, Begg and Egger tests were performed. The result of the Begg method was nonsignificant (*P* = .350), suggesting no significant publication bias, as shown in Figure 6. The result of the Egger method was significant (*P* < .001), suggesting the existence of publication bias, as shown in Figure 7. The publication bias may be due to the fact that articles with negative results are not easy to publish, because the literature was all published in Chinese, which is subject to language bias, and because fewer study subjects were included in some of the articles.

## 4. Discussion

For this meta-analysis, the screening process and criteria were developed by searching domestic and international medical databases and strictly following the PRISMA principles. Ultimately, 11 RCTs involving 1142 patients were included in this study; a total of 571 patients were included in the blade needle group, with 510 effective cases of blade needle therapy, and 571 patients were included in the conventional acupuncture group, with 413 effective cases of conventional acupuncture. Meta-analysis showed OR = 3.61, 95% CI [2.56–5.10], *P* < .00001, indicating that blade needle therapy is better than conventional acupuncture for the treatment of KOA (*P* < .05). Altogether, the findings suggest that blade needle treatment is preferred in the treatment of KOA in clinical practice.

**Figure 8.**
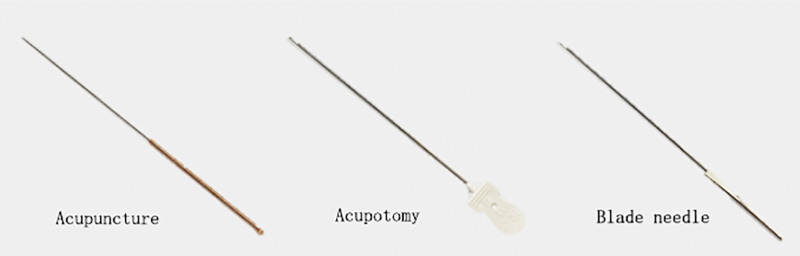
Comparison of the structure of acupuncture, acupuncture knives and blade needle state that the review was not registered state that a protocol was not prepared. Note: The supplementary document “PubMed” is the search format used for this article on the PubMed website, http://links.lww.com/MD/H164

Currently, there is no consensus on the best treatment for KOA, and there is no curative treatment at home or abroad. Western medical treatment mainly includes nonsteroidal antiinflammatory drugs, antirheumatic drugs, glucocorticoids, intraarticular injection drugs and surgical treatment.^[40]^ Although these treatments have achieved good clinical results, adverse effects such as gastrointestinal reactions and liver and kidney injury should not be ignored. Traditional Chinese medicine treatments for knee osteoarthritis mainly include internal treatment, external treatment, acupuncture therapy, tui-na, etc. Chinese medicine treatment not only relieves clinical symptoms and improve the function of the knee joint of patients but also delays the destruction of joint cartilage and prevents the rapid progression of knee osteoarthritis.^[41]^ To date, blade needle therapy has been widely used in clinical practice for the treatment of knee arthropathies, degenerative spinal diseases, frozen shoulder, muscle strain and cutaneous nerve entrapment and has achieved certain efficacy.^[42]^ Blade needles cut a small amount of tense muscle fibers or fascia to reduce the tension stress of the tissue, decrease the abnormal adhesions between the tissue muscle fascia, and mitigate the compression effect of the diseased tissue on blood vessels, nerve bundles, and bone fiber canals. Compared with conventional acupuncture treatment, the cutting and release action of blade needle therapy is more advantageous for improving joint pain and functional issues. Blade needle therapy is implemented under the guidance of meridian tendon theory in combination with information from Western medicine and taking into account the body’s structural mechanics and anatomy, among other disciplines. Needles are placed through the point of meridian tendon lesions to unblock peeling, release adhesions, restore the mechanical balance of the joint, improve blood flow and lymphatic circulation in the joint, and promote local tissue metabolism, thereby correcting the joint and making it more tender with the goals of improving joint pain, movement and function and ensuring the continuity of the therapeutic effect.^[43]^ As a minimally invasive medical technique, this therapy presents a unique approach to improving the balance of forces within the knee joint and is effective at relieving clinical symptoms. A comparative diagram of the structures of acupotomy, acupuncture and blade needle therapy is shown in Figure 8.

Through this study, we expect to provide some evidence for the clinical effectiveness of blade needle in the treatment of KOA, and to dispel the misconceptions or fears of the public about blade needle. Because the body and blade of the blade needle are smaller than those used for acupuncture,^[44]^ the blade needle causes less trauma to the patient during treatment and is less painful and easier to operate, and the standardized approach to blade needle therapy can prevent the dissociation of nerves and tendons and is easy to master^[45]^, while it is more acceptable to patients and more clinically developed and popular because it is less expensive than surgical and other treatments.

However, only 5 of the 11 articles included in this study mentioned a specific randomized method, and none of the articles mentioned blinding or provided information on the application procedure, which might be due to a lack of attention to these details. In terms of adverse events, none of the studies reported whether adverse reactions were observed, and the safety of blade needle treatment for KOA has not been evaluated. It is hoped that more details on its safety will be reported in future RCTs. The inclusion of literature in Chinese and the difficulty of publishing negative trials may have resulted in some publication bias.

As all the included trials measured efficacy only at the end of treatment, no comparison can yet be made regarding the long-term efficacy of blade needle therapy and conventional acupuncture for KOA. There are also some limitations to this study. The observation indicators and evaluation methods were not uniform across the studies, so it was impossible to conduct meta-analyses of the measures; thus, some specific indicators could not be analyzed. Additionally, the quality of the included literature was variable for the following reasons: (i) Three articles failed to mention diagnostic criteria. (ii) The descriptions of the randomization methods were not specific, with only 5 articles mentioning specific randomization methods. (iii) Allocation concealment was not mentioned in any of the studies, although it could be inferred from the original text in 2 studies. (iv) Considering the characteristics of the blade needle treatment itself, the blinding of patients as well as implementers could not be achieved; however, blinding could have been achieved in some of these sessions if the researcher had considered the experimental design more thoroughly. (v) Only 1 article mentioned follow-up, so the long-term efficacy of blade needle therapy for KOA remains unclear. (vi) The search included only a selection of commonly used databases in English and Chinese, which probably resulted in incomplete searches. However, through this study, it is expected that relevant investigators will be motivated to participate in more standardized training on clinical trial methodology, with the aim of reporting more relevant low-risk, high-quality studies in the future and providing stronger evidence for assessing the clinical efficacy of blade needle treatment for KOA.

This study found that few articles reported measures of the efficacy of blade needle therapy for KOA treatment, such as Visual Analogue Scale (VAS) scores, WOMAC scores, or joint mobility scores; therefore, it is difficult to standardize these studies. Moreover, the follow-up period for blade needle therapy for KOA treatment has been relatively short, so in the future, observational studies of its long-term efficacy need to be strengthened to further clarify the effectiveness of this therapy.

## Author contributions

Xunlong Yin: coordinated planning, reviewed literature, screened data, did data analysis, wrote the article

Yuan Liu: rational division of labor, checking data, guiding writing the essay

Wu Liu: screening literature, checking data

Wei Liang: sifting literature, screening data, doing data analysis

Qingyong Liang: screening literature, checking data

## Supplementary Material



## References

[1] Felson DT Lawrence RC Dieppe PA. Osteoarthritis:new insights.Part1:the disease and its risk factors. Ann Intern Med. 2000;133:635–46. 11033593 10.7326/0003-4819-133-8-200010170-00016

[2] Peat G McCarney R Croft P. Knee pain and osteoarthritis in older adults:a review of community burden and current use of primary health care. Ann Rheum Dis. 2001;60:91–7. 11156538 10.1136/ard.60.2.91 PMC1753462

[3] Dillon CF Rasch EK Gu Q. Prevalence of knee osteoarthritis in the United States: arthritis data from the third national health and nutrition examination survey 1991-1994. J Rheumatol. 2006;33:2271–9. 17013996

[4] Tang X Wang S Zhan S. The prevalence of symptomatic knee osteoarthritis in china:results from the china health and retirement longitudinal study. Arthritis Rheumatol. 2016;68:648–53. 26474054 10.1002/art.39465

[5] Neogi T Zhang Y. Epidemiology of osteoarthritis. Rheum Dis Clin North Am. 2013;39:1–19. 23312408 10.1016/j.rdc.2012.10.004 PMC3545412

[6] Wang B Xing D Dong S. Systematic evaluation of the epidemiology and disease burden of knee osteoarthritis in China. Chin J Evid -Based Med. 2018;18:134–42.

[7] Chen Y Zou Q Ji S. Meta analysis of the efficacy and safety of warm acupuncture treatment in knee osteoarthritis. Chin Ethnic Folk Med. 2019;28:45–50.

[8] Liu X. Current research status of TCM for the treatment of knee osteoarthritis. Bone. 2012;24:3–7.

[9] Qiu H. Overview of knee osteoarthritis treatment. Arthritis Rheumatol. 2013;2:59–63.

[10] Cao G Shen H An S. Complications and efficacy analysis of artificial total knee replacement in elderly patients. N Engl J Med. 2015;9:50–4.

[11] Zhu Z. Analysis of total knee replacement complications and knee function evaluation in obese patients. J China Med Univ. 2010;9:1–36.

[12] Hussain OT Sah S. Prospective comparison study of one-year outcomes for all titanium total temporomandibular joint replacements in patients allergic to metal and cobalt–chromium replacement joints in patients not allergic to metal. Br J Oral Maxillofac Surg. 2013;52:34–7. 23522619 10.1016/j.bjoms.2013.02.014

[13] You W Lin X Yuan H. Clinical study of poor incision healing after total knee replacement. chin J Bone Jt Injury. 2014;29:391–2.

[14] Nabil AE Joseph RR Wandtke ME. Systematic review of periprosthetic tibia fracture after total knee arthroplasties. World J Orthop. 2015;6:649–54. 26396942 10.5312/wjo.v6.i8.649 PMC4573510

[15] Gehrke T Alijanipour P Parvizi J. The management of an infected total knee arthroplasty. Bone Joint J. 2015;97:9–20. 26430083 10.1302/0301-620X.97B10.36475

[16] Xian Han X Cheng S. Progress in the external treatment of knee osteoarthritis. Arthritis Rheumatol. 2017;6:72–72.

[17] Wang Y Wang N Qiao Y. . Clinical study of acupotomy for knee osteoarthritis. Tradit Chin med. 2020;52:116–9.

[18] Zhou W Yang Y Chen Z. Overview of clinical studies of acupotomy for knee osteoarthritis. J Guiyang Tradit Chin Med. 2018;40:76–9.

[19] Zhao M Bai Y Zhang Y. Research progress of small acupotomy for knee osteoarthritis. Hebei TCM. 2017;39:1908–12.

[20] Tian H Li Z Zhang H. Progress in acupuncture treatment for knee osteoarthritis. J Tradit Chin Med. 2019;21:217–9.

[21] Cheng J Ye Z. Progress in acupuncture and moxibustion treatment of knee osteoarthritis in the last five years. CHMA. 2020;22:261–4.

[22] Chen L Chen B Lin A. Progress in the clinical research of traditional Chinese and western medicine therapy for knee osteoarthritis. J Tradit Chin Med. 2020;47:203–6.

[23] Li J Li YX Luo LJ. The effectiveness and safety of acupuncture for knee osteoarthritis:An overview of systematic reviews. Med (Baltim). 2019;98:e16301. 10.1097/MD.0000000000016301 PMC6641846 31305415

[24] Xu Q Qiu H Zhu Z. Acupotomy for knee osteoarthritis: a systematic review protocol . Med (Baltim). 2019;98:e17292. 10.1097/MD.0000000000017292 PMC6775403 31574849

[25] Feng W Ding M. Progress in acupuncture for knee osteoarthritis in the past 5 years. Clin J Acupunct. 2019;35:81–4.

[26] Tian J. Blade needle therapy(1). The historical origin of blade needle therapy. Chin Acupunct. 2005;25:139–40.

[27] Tian J. Blade needle therapy(2). Theoretical basis and mechanism of action of blade needle therapy. Chinese Acupuncture. 2005;25:201–2.

[28] Tian J. Blade needle therapy(3). Indications, contraindications and operational essentials of blade needle therapy. Chin Acupunct. 2005;25:285–6.

[29] Huang Y Nie B. Efficacy observation of tendon point blade needle loosening in knee osteoarthritis. Rehabilit Med. 2015;6:17–8.

[30] Xu Y Yue S Li J. Observation of the clinical effect of tendon point blade needle loosening in knee osteoarthritis. Clin misdiagnosis mistreat. 2019;32:50–4.

[31] Du S Guo Z Kong Q. Clinical study of arc-Blade needle in the treatment of osteoarthritis of the knee. Chin J Tradit Chin Med. 2018;33:1657–60.

[32] Zheng L Yu J Zhang Z. Clinical observation of oblique round blade needle in the treatment of knee majesty osteoarthritis. Chin TCM emergency. 2014;23:2362–3.

[33] Duan J. Discussion on the clinical effect of the treatment of knee osteoarthritis with a blade needle. Contemp Med t. 2014;12:34–5.

[34] Xiang B Zhai H. Clinical study of blade needle treatment for knee osteoarthritis. Electron J Gen Med. 2019;6:141–141.

[35] Chen C. Clinical observation of blade needle treatment for knee osteoarthritis. Chin Ethnic Folk Med. 2018;27:79–81.

[36] Yang M Li S. Clinical observation of blade needle for knee osteoarthritis. Chin Folk Therapy. 2020;28:19–20.

[37] Li L. Comparative study of blade needle and acupuncture treatment for knee osteoarthritis. J Tradit Chin Med. 2013;22:34–5.

[38] Wu M. The effect of blade needle treatment in knee osteoarthritis and its effect on biomechanics of X line. J Pract Pain st. 2011;7:261–3.

[39] Zhang L. Evaluation of the clinical application of arc-edged needle on knee osteoarthritis. Health Care Guide. 2018;37:84–84.

[40] Lin LL Tu JF Shao JK. Acupuncture of different treatment frequency in knee osteoarthritis: a protocol for a pilot randomized clinical trial. Trials. 2019;20:423. 31296249 10.1186/s13063-019-3528-8 PMC6625113

[41] Liu Q Hong GY Hu WJ. Advances in the treatment of osteoarthritis of the knee. Med Rev. 2015;21:474–6.

[42] Li T Xu S. Clinical efficacy evaluation of blade needle with strong fumigation on knee osteoarthritis. Rehabil Med. 2020;11:79–81.

[43] Qiu F Zhang X. Progress in the external treatment of knee osteoarthritis by“menstrual tendon theory”. J Clin Acupunct. 2018;34:87–9.

[44] Wang F. Clinical study of Blade needle for knee osteoarthritis. Nanning. 2019;1:1–54.

[45] Guo B Yu S Hu Y. Progress in the clinical application and mechanism of action of the blade needle. J Cosmet Dermatol. 2016;32:210–2.

